# Population dynamics of the glacial relict amphipods in a subarctic lake: role of density‐dependent and density‐independent factors

**DOI:** 10.1002/ece3.8260

**Published:** 2021-11-09

**Authors:** Alexey A. Maximov

**Affiliations:** ^1^ Zoological Institute Russian Academy of Sciences St Petersburg Russia

**Keywords:** climate variation, density dependence, long‐term monitoring, *Monoporeia affinis*, population dynamics, subarctic lakes

## Abstract

Relative role of intrinsic density‐dependent factors (such as inter‐ and intraspecific competition, predation) and extrinsic density‐independent factors (environmental changes) in population dynamics is a key issue in ecology. Density‐dependent mechanisms are considered as important drivers of population dynamics in many vertebrate and insect species; however, their influence on the population dynamics of freshwater invertebrates is not clearly understood. In this study, I examined interannual variations in the abundance of the glacial relict amphipod *Monoporeia affinis* in a small subarctic lake based on long‐term (2002–2019) monitoring data. The results suggest that the population dynamics of amphipods in the lake is influenced by the combined effects of both intrinsic and extrinsic factors. The reproductive success of amphipod cohorts was inversely related to its initial abundance, indicating it is influenced by density‐dependent factors. *M*. *affinis* recruitment was negatively correlated with population density and near‐bottom temperature but positively correlated with food availability, which is defined as the concentration of chlorophyll *a*. Multiple regression with chlorophyll, temperature, and abundance of parent cohort as independent factors explained about 80% of the variation in the reproductive success of amphipods. The negative correlation between amphipod recruitment and water temperature indicates that the current climate conditions adversely affect the populations of glacial relict amphipods even in cold‐water lakes of the subarctic zone. Results of this study can be useful in environmental assessments to separate population oscillations connected with density‐dependent mechanisms from human‐mediated changes.

## Graphical abstract

Long‐term (2002–2019) variations in the abundance of the glacial relict amphipod *Monoporeia affinis* in a small subarctic lake were examined. The results suggest that population dynamics of amphipods is influenced by the combined effects of both intrinsic (population density) and extrinsic (water temperature, food availability) factors.

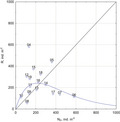

## 1 INTRODUCTION

Understanding the causes of year‐to‐year fluctuations in species abundance is a key issue in ecology. Particularly, the relative role of intrinsic density‐dependent factors (such as inter‐ and intraspecific competition, predation) and extrinsic, as a rule density‐independent, factors (environmental changes) in population dynamics was debated for almost a century. Although nowadays it is generally agreed that both intrinsic and extrinsic factors are important (Bjørnstad & Grenfell, 2001; Stenseth et al., 2002; Stenseth, 2007), there are advocates of categorical views. For example, some authors disclaim density‐dependent regulation of abundance (Sakuramoto, 2015; White, 2008), while others believe that the population dynamics of many species is indeed regulated by density‐dependent mechanisms (Sibly et al., 2005).

A strong effect of density‐dependent regulation is widely accepted in vertebrate research especially of charismatic and economically important species such as ungulates and commercial fishes (Forchhammer et al., 2002; Lorenzen & Enberg, 2002). Also, density‐dependent factors are believed to be of key importance in regulating insect populations (Nowicki et al., 2009; Ouyang et al., 2014; Sibly et al., 2005). Density‐dependent processes in populations of freshwater invertebrates are still poorly understood. As a rule, it is considered that invertebrates are influenced mainly by environmental conditions directly or indirectly (as a result of climate‐induced variations in primary production and food availability) (Jyväsjärvi & Hämäläinen, 2014; Maximov et al., 2021).

Macroinvertebrates, especially sedentary and long‐life benthic species, are widely recognized as an indicator of change in environmental conditions in both freshwater and marine monitoring programs (Perus et al., 2007; Rosenberg & Resh, 1993). It highlights the need to understand the mechanisms of population change in indicator species, and in particular, to quantitate the role of both density‐independent (including human activity) and density‐dependent factors. Understanding population change is also essential for conservation of imperiled invertebrate species.

In this study, I examined the population dynamics of the glacial relict amphipod *Monoporeia affinis* (fam. Pontoporeiidae) in a small subarctic lake based on long‐term monitoring data. Amphipoda is a well‐established and long‐recognized indicator of good ecosystem health for assessing the ecological status of aquatic environments (Dauvin, 2018). In particular, pontoporeiid species are known to be highly sensitive to climate warming and different local environmental disturbances like eutrophication, pollution, acidification (Demandt et al., 2012; Goedkoop, 2006; Maity et al., 2013). In the Baltic Sea, *M*. *affinis* is used as a bioindicator to monitor benthic community ecology and biological effects of contaminants (Löf et al., 2016; Sundelin & Eriksson Wiklund, 1998).

Glacial relict amphipods from fam. Pontoporeiidae are a key element in the profundal benthic community of many deep northern lakes. They play a major part in the flow of energy and nutrients through the food web forming an important link between pelagic production and fish (Gardner et al., 1990; Johnson & Wiederholm, 1992; Nalepa et al., 2009a). The population dynamics of these species can, therefore, have cascading effects across multiple trophic levels (Nalepa et al., 2009b; Pothoven et al., 2011). Currently, glacial relict amphipods are experiencing a global decline attributing to the degradation of their habitats by eutrophication, hypoxia, climate changes, biological invasions, and other human‐caused phenomena (Eriksson Wiklund & Andersson, 2014; Kalinkina et al., 2016; Watkins et al., 2013). However, the mechanisms underlying such a decline are not fully understood.

The worldwide declines together with high vulnerability and important food web role highlight the need to examine the drivers of the population dynamics of pontoporeiid amphipods. Here I present an analysis of interannual variations in the abundance of the population of *M*. *affinis* that exists in nearly pristine oligotrophic northern lakes. The absence of a local anthropogenic impact provides an opportunity to assess the importance of natural extrinsic as well as intrinsic factors for amphipod population. The aims of the study were: (1) to examine the presence of any density dependence, (2) to evaluate the effects of environmental variability, including variations in pelagic productivity, on the population dynamics of amphipod, and (3) to assess the relative importance of density‐dependent versus density‐independent factors. Water temperature, oxygen concentrations, and food availability are recognized as the most important environmental factors limiting the abundance of *M*. *affinis* (Goedkoop, 2006; Gordeev, 1952; Sarvala, 1986). There are also field‐based and experimental evidence that show the effects of density‐dependent processes on *M*. *affinis* populations, especially from the Baltic Sea (Leonardsson, 1994; Sarvala, 1986; Wenngren & Ólafsson, 2002). Oxygen level cannot be the decisive factor here, because at the study site oxygen concentrations were more than 10 mg/L. In this paper, I examine the impact of water temperature, food, and intraspecific competition on amphipod reproductive outcome. The annual mean chlorophyll *a* concentration was used as an indicator for pelagic primary production and food availability.

## 2 MATERIALS AND METHODS

### 2.1 Study lake

Lake Krivoe is a small (0.5 km^2^) oligotrophic lake located at the northernmost (30 km below the Polar Circle) part of the Republic of Karelia (northwest of the Russian Federation) in the immediate vicinity of the White Sea coast. The mean and maximum depths of the lake are 12 and 32 m, respectively. The catchment area is unpeopled and mostly covered by bare rocks with lichens and mosses and sparse growth of pine trees. Lake Krivoe is dimictic. Ice‐covered period lasts from November to May in the lake. The bottom waters of the lake are usually well‐saturated (60–98%) by oxygen. Only in local deep (depth 30 m), hypoxic conditions are registered toward the end of the ice‐covered period.

The study site (66°20.774′N and 33°37.77′E) is situated in the sublittoral zone at a depth of 8.5 m. Near‐bottom temperature varies from 2 to 3°C during ice‐covered period to 10–13°C in late August and September. Bottom sediment is soft brown silt.

### 2.2 Study species

**FIGURE 1:**
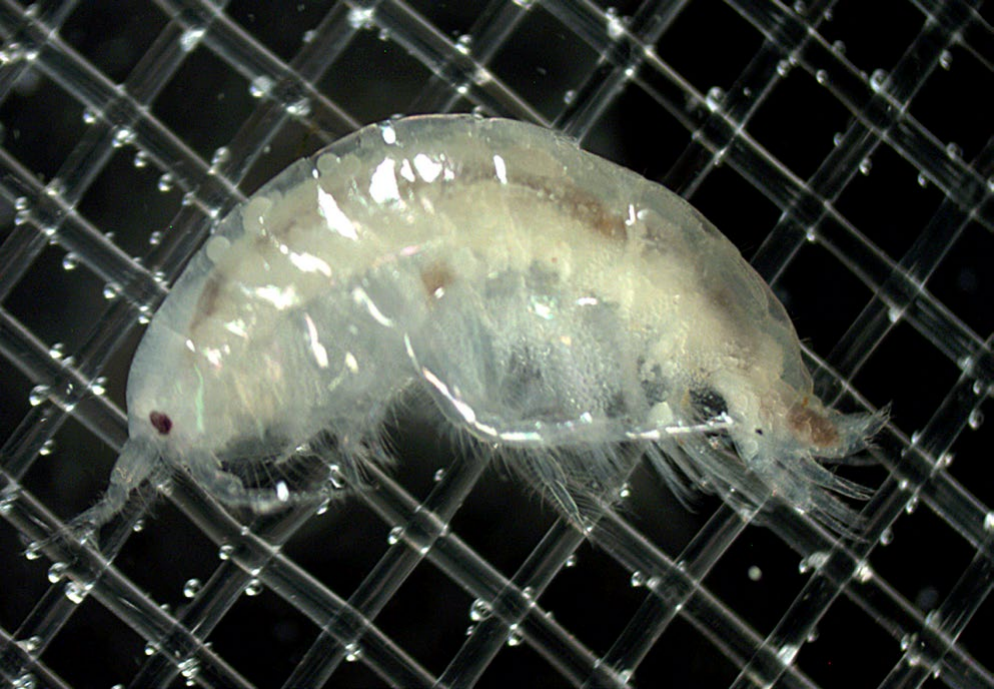
The glacial relict amphipod *Monoporeia affinis* (Lindström) (fam. Pontoporeiidae) from Lake Krivoe. The diagonal size of screen mesh is 1 mm

The semelparous amphipod *M*. *affinis* (Figure 1) is a winter breeder. The adults copulate from December to January. Males die immediately after copulation. The ovigerous females carry their embryos in marsupium and die after releasing their young in March–April. The duration of the life cycle of amphipods depends on their growth rate and varies from 1 to 4 years. In some habitats and/or years, amphipods have no time to reach a threshold size in order to reproduce; thus, they remain immature throughout the first 1–3 years of their life (Leonardsson, 1988; Sarvala, 1986). Overwintered individuals live during the following summer and autumn up to the next reproduction period in winter. Because of the short period of recruitment, different cohorts can be clearly distinguished on histograms of length‐frequency distribution. In Karelian lakes, *M*. *affinis* generally reproduces during the second year of its life (Gordeev, 1952).

Lake Krivoe is characterized by a simple food web. There are only three fish species in the lake. Two of them perch, *Perca fluviatilis*, and vendace, *Coregonus albula*, feed mainly on macrofauna including *M*. *affinis* (Berezina et al., 2018, 2021).

### 2.3 Field methods

Materials were collected at one sublittoral site from 2002 to 2019 mainly during the ice‐free period (late May–October) as a rule, four to five times a season. Benthic samples were taken by standard Van Veen grab (area 0.025 m^2^) in five replicates and were sieved through a screen with mesh sizes of 0.25 mm. In the laboratory, all macroinvertebrates were sorted and preserved in 70% ethanol. To estimate the age structure of *M*. *affinis* population, the body length (distance between rostrum and telson base) of the preserved amphipods was later measured to the nearest 0.1 mm. Altogether about 4000 (that is 50 individuals per sampling occasion) amphipods were measured. The age groups were separated subjectively based on histograms of length‐frequency distribution.

Near‐bottom (7–8 m) temperature during the ice‐free period was measured on a biweekly or monthly basis. The mean temperature was calculated by integrating these data. The annual mean chlorophyll *a* concentration was used as an indicator for phytoplankton development and food availability for macrozoobenthos. The concentration of chlorophyll from 2010 was obtained from monthly integral water column samples (0–7 m) collected by Ruttner bottle from May to October. The earlier (2002–2009) data are average annual values accepted from Maximov et al. (2021). In both cases, the concentration of chlorophyll *a* was measured using standard technique (UNESCO, 1966).

### 2.4 Time series analysis

The time series obtained were analyzed with partial correlation and multiple regression analyses (TIBCO Software Inc., 2020). By the use of partial correlation analyses, I first examined the relationship between recruitment (number of offspring) and different extrinsic and intrinsic variables. The following variables were used: (a) mean water temperature during one (*T*_1_) and two (*T*_2_) years preceding the new cohort appearance; (b) mean concentration of chlorophyll *a* during one (Chl_1_) and two (Chl_2_) years preceding the new cohort appearance; (c) abundance of parent cohort in ages 0+ (*N*_P0+_) and 1+ (*N*_P1+_); (4) abundance of older (*N*_old_) and younger (*N*_young_) cohorts which co‐occurred and potentially competed with parent generation, respectively, in the first and second years of life. Then, significant relations were tested by a multiple regression analysis. Before this analysis, potential multicollinearity between predictor variables was checked with the Pearson correlation test. There was no significant pairwise relationships (*p *> .1). The significance of each of the explanatory variables was also examined using Akaike Information Criterion (AIC). A lower AIC value indicates a better model fit. The addition of a parameter is considered significant if the AIC is decreased by more than 2 (Burnham & Anderson, 2002). Finally, I modelled the influence of density on the reproductive outcome of amphipod by using the stock‐recruitment function of Ricker (1954), the classical model most widely used to describe dynamics of fish populations. This simple population model (so‐called reproduction curve) expresses the ratio between sizes of parental (*N*_P_) and filial (recruitment, *R*) generations at the same stage of the life cycle:

$$R=N_{P\;}e^{a\left(1‐\frac{N_{P}}{b}\right)}$$

Coefficients *a* and *b* were determined using a linear form of this function (Ricker, 1975):

$$\ln R‐\ln N_{P}=a‐a/bN_{P}$$

## 3 RESULTS

### 3.1 Environmental factors

**FIGURE 2:**
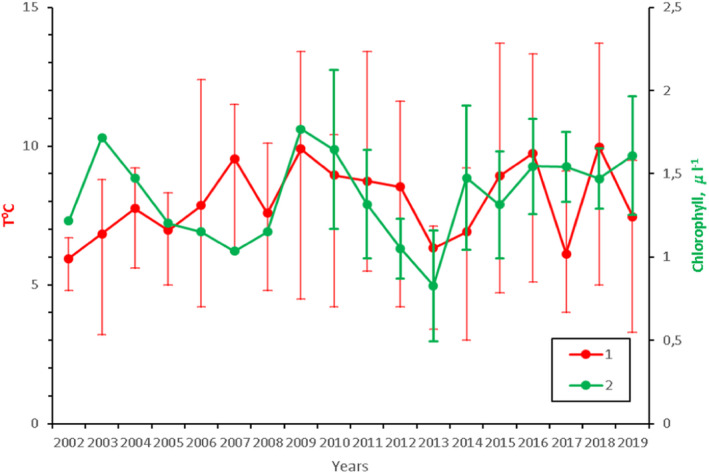
Changes in mean water temperature and chlorophyll *a* concentration at study site during open‐water period (May–October) in 2002–2019. (1) Near‐bottom (7–8 m) temperature (±range). (2) Concentration of chlorophyll *a* (0–7 m). The mean concentration (±standard error) was calculated by pooling water samples taken at study site during open‐water period. Earlier published data of 2002–2009 are presented without standard errors because seasonal data series are not available

Interannual variations in near‐bottom water temperature and chlorophyll *a* concentration are presented in Figure 2. The mean temperature varied within a relatively narrow limit between 6.0 and 10.0°C. The lake water is typified by low primary production. The highest concentrations of chlorophyll *a* (up to 3 *µ*g/L) were recorded in June. The mean annual values varied from 0.83 to 1.77 and averaged 1.36 *µ*g/L.

### 3.2 Macrobenthic community

**FIGURE 3:**
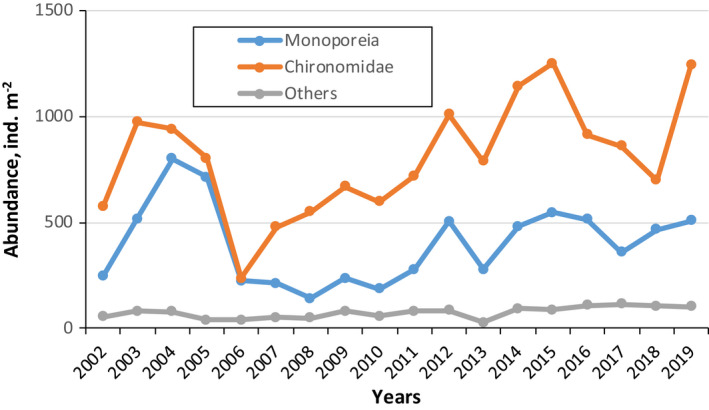
Changes in mean annual abundance (ind./m^2^) of main macrobenthic taxa at study site in 2002–2019

Macrozoobenthos at the study site was strongly dominated by amphipod *M*. *affinis* and chironomids (Figure 3). These two taxa formed more than 90% of the abundance and 80% of the biomass of the whole macrozoobenthos. *M*. *affinis* contributes 31% of total abundance and 60% of the total biomass. There were no major differences in the composition of macrozoobenthos across the years. There was a significant positive correlation between mean annual values of abundance (*R* = 0.672, *p *< .01) and biomass (*R* = 0.854, *p *< .001) of both main taxa.

### 3.3 Population dynamics of *Monoporeia affinis*

**FIGURE 4:**
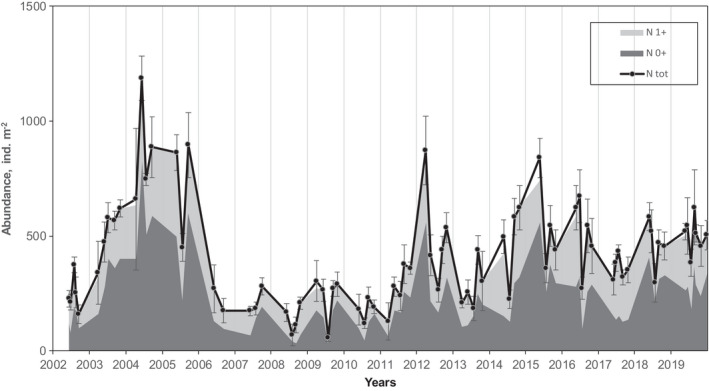
*Monoporeia affinis* population abundance (±standard error) from June 2002 to December 2019. *N* 0+ and *N* 1+ indicate abundance of age group 0+ and 1+, respectively.

The study population of *M*. *affinis* demonstrated considerable intra‐ and interannual fluctuations. (Figures 3 and 4). Lowest abundance occurred in late summer 2008 and 2009 (56 and 70 ind./m^2^, respectively), and highest (1187 ind./m^2^) in June 2004. Mean annual abundance exhibited long‐term variations with two peaks in 2004 and mid‐2010 (Figure 3).

### 3.4 Dynamics of different age groups

**FIGURE 5:**
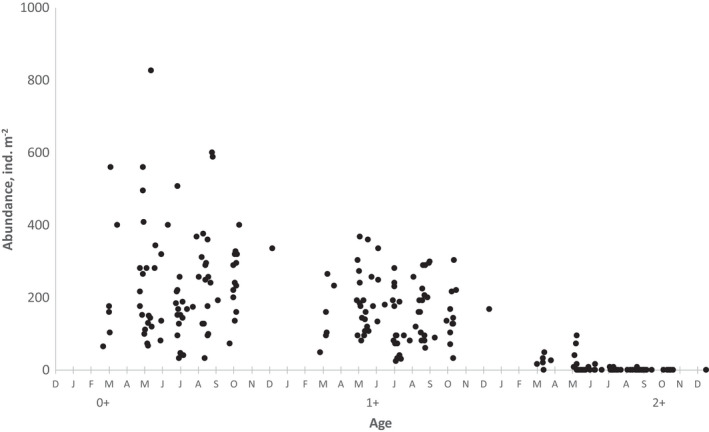
Age‐related dynamics of *Monoporeia affinis* abundance

**FIGURE 6:**
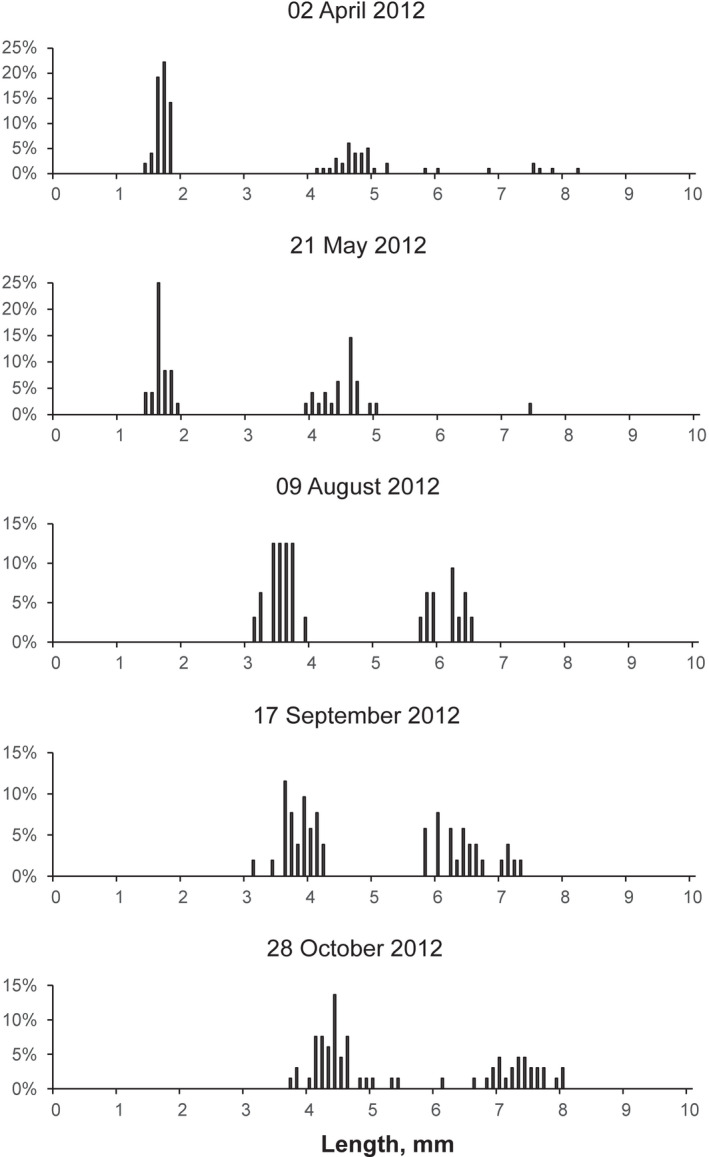
Seasonal dynamics of length‐frequency distribution of a population of *Monoporeia affinis*. Data of 2012 are presented as an example

During the study period, the young amphipods were released in late March and early April. Basically, the abundance of each cohort decreased gradually with considerable scatter until almost complete disappearance in the spring following reproduction at the second year of life. Only a few individuals remain immature through the second winter and live up to the third year (age group 2+) (Figure 5). Thus, two distinct age groups (0+ and 1+) were presented in summer and autumn because almost all older individuals died during spring (Figure 6). The abundance of different cohorts varied markedly across years. During each year, the abundance of both age groups fluctuated within wide limits, but maximum values were generally observed in spring or early summer (Figure 4).

Due to the great intra‐annual (and/or among samples) data variability, mean annual numbers were used in statistical analyses which were estimated by averaging abundances of each age group throughout the whole year. Analysis revealed that population recruitment was inversely related with the initial abundance of the parent cohort and co‐occurred older cohort (i.e., abundance of 0+ and 1+ groups two years ago, respectively) (Table 1). At the same time, there was no significant correlation between recruitment and abundance values in the year preceding the appearance of young (one year ago). Also, variations in the recruitment were closely related with studied environment factors during both years preceding reproduction. There was a significant positive partial correlation with the concentration of chlorophyll *a* and negative correlation with water temperature (Table 1).

**TABLE 1:** Partial correlation between recruitment (*R*) and different variables describing abundance (*N*_P0+,_ *N*_old_, *N*_tot‐2_, *N*_P1+_, *N*_young_, *N*_tot‐1_), concentration of chlorophyll *a* (Chl_1_ and Chl_2_), and water temperature (*T*_1_ and *T*_2_). Marked correlations are significant at *p < .05, **p < .01, ***p < .001

Relation	Abundance	Chlorophyll	Temperature
*R*: *N*_P0+_ – Chl_2_ – *T*_2_	−0.578*	0.723**	−0.852***
*R*: *N*_old_ – Chl_2_ – *T*_2_	−0.663**	0.783***	−0.880***
*R*: *N*_tot−2_ – Chl_2_ – *T*_2_	−0.615*	0.747**	−0.863***
*R*: *N*_P1+_ – Chl_1_ – *T*_1_	0.093	0.576*	−0.54*
*R*: *N*_young_ – Chl_1_ – *T*_1_	0.511	0.616*	−0.628*
*R*: *N*_tot−1_ – Chl_1_ – *T*_1_	0.363	0.583*	−0.579*

Notes

*N*_P0+,_ *N*_old_ and *N*_tot‐2_ are respectively abundance of 0+ (parent cohort), 1+ (older cohort) groups and total population 2 years ago. *N*_P1+_, *N*_young_, *N*_tot‐1_ are respectively abundances of 1+ (parent cohort) and 0+ (younger cohort) groups and total population 1 year ago. Chl_1_ and *T*_1_ Chl_2_ are respectively mean concentration of chlorophyll *a* and temperature of near‐bottom water during 1 year preceding the recruitment period. Chl_2_ and *T*_2_ are respectively mean concentration of chlorophyll *a* and temperature of near‐bottom water during the 2 years preceding the recruitment period.

The multiple regressions with chlorophyll, temperature, and abundance values 2 years back (*N*_P0+,_ *N*_old_, and *N*_tot‐2_) as independent factors explained about 80% of the variation in the population recruitment:

*R* = 901–0.347·*N*_P0+_ + 332·Chl_2_ – 128·*T*_2_ (*R* ^2^ = .769; *p *< .0004)
*R* = 1011–0.767·*N*_old_ + 366·Chl_2_ – 142·*T*_2_ (*R* ^2^ = .806; *p *< .00014)
*R* = 942–0.27·*N*_tot‐2_ + 343·Chl_2_ – 132·*T*_2_ (*R* ^2^ = .784; *p *< .00027)

**FIGURE 7:**
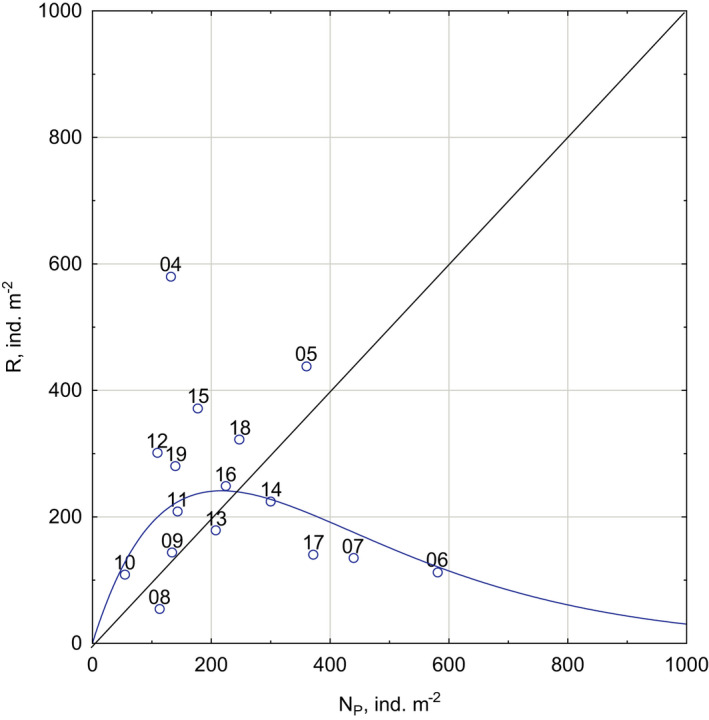
Abundance of parent (*N*_P)_ and filial (*R*) cohorts in age 0+. The fitted functions represent Ricker stock‐recruitment model $R=N_{P}e^{1.1043\left(1‐\frac{N_{P}}{240}\right)}$. The thin solid straight line is bisectrix of quadrantal angle. Labels are years of recruitment

The best one‐parameter regression model with lowest AIC (198.3) was with water temperature. The addition of chlorophyll concentration considerably improved fit (AIC = 188.9). The best performing model included temperature, chlorophyll, and abundance values. The AIC decreased by more than 2 as a result of adding abundance values, indicating that the fits were significantly improved. At the same time, according to AIC (184.9‒185.5) there were practically no differences between fits with different abundance values (*N*_P0+,_ *N*_old_, and *N*_tot‐2_). The relationship between the reproductive outcome of the *M*. *affinis* cohort and its initial abundance was significantly described by the Ricker model (Figure 7):

$$R=N_{P0}+e^{1.1043\left(1‐\frac{N_{\text{P0 +}\;}}{240}\right)}\left(p=.006\right)$$

## 4 DISCUSSION

The results of this study suggest that the population dynamics of *M*. *affinis* in Lake Krivoe is influenced by the combined effects of both intrinsic (population density) and extrinsic (temperature and concentrations of chlorophyll *a*) factors. Reproductive success of the cohort was inversely related with its initial abundance and abundance of older age group, indicating density dependence.

In contrast, there was no significant correlation with abundance of younger cohort. This result agrees with experimental studies that growth and mortality in juvenile *M*. *affinis* are dependent on the density of adults, but juveniles do not affect adults (Elmgren et al., 2001; Hill, 1992; Wenngren & Ólafsson, 2002). The descending right limb of the reproduction curve begins above a bisectrix of quadrantal angle (Figure 7). This type of dependence between parental and filial generations should result in cyclic oscillations in population size (Ricker, 1954). However, the Ricker model predicts that these cycles are damped and should eventually disappear because of the flat slope (lesser than 45°) of the right limb. Therefore, the observed considerable interannual fluctuations seem impossible without external environmental influence.

Temperature and food conditions are crucial for the recruitment success of lake populations of *M*. *affinis* (Goedkoop & Johnson, 2001; Johnson & Wiederholm, 1992). In Lake Krivoe, abundance of offspring was negatively correlated with thermal conditions during two preceding years (i.e., through life of parental cohort). *M*. *affinis* is a cold stenothermal glacial relict species, and it is sensitive to high water temperature. In the Baltic Sea this amphipod is known to escape from warm shallower areas toward colder deep waters during thermal stratification in late summer (Sarvala, 1986). At the study site, near‐bottom temperature (Figure 2) was lower than optimum, 12–15°C, as claimed for Karelian lakes by Kaufman (Kaufman, 2001) based on thermopreferendum data. The annual means during the study period were below 10°C (Figure 2). However, studies from the Baltic Sea have shown that temperature conditions that are not detrimental to adults could severely affect the gonads and embryos (Eriksson Wiklund & Sundelin, 2001; Reutgard et al., 2014). In the sublittoral zone of Lake Krivoe, the period of gonad development coincides with the maximum water temperatures in early September. Thus, temperature stress during maturation can cause serious harm to the reproductive success of the species. Also, elevated temperature may indirectly affect the abundance of amphipods through deterioration of nutritional condition and (or) intensity of fish predation. Food shortage for amphipods is more probable in warmer water because of higher metabolic rate and energy losses at high temperature. In Lake Krivoe, amphipods are consumed by perch (*Perca fluviatilis*) and vendace (*Coregonus albula*) (Berezina et al., 2018). These fish switch to feeding on *M*. *affinis* in late August–October when deep waters warm up during autumn mixing. It could be that with increased temperature, fish extend their depth range, leading to greater predation pressure on zoobenthos.

Food availability is the main factor limiting animal populations (White, 2008). Benthic invertebrates are strongly dependent on sedimentation of organic matter from pelagial zone. Temporal and spatial variations in pelagic primary production have a profound effect on the food availability for macrozoobenthos in lakes (Hayden et al., 2017; Jyväsjärvi et al., 2012). In Swedish lake Vänern, the interannual fluctuations of *M*. *affinis* abundance were highly correlated with spring maximum diatom biovolume at a 1‐year lag (Goedkoop & Johnson, 2001; Johnson & Wiederholm, 1992). In Lake Krivoe, year‐to‐year dynamics of littoral communities seems to follow the variations in chlorophyll *a* level with maximum biomass values 1–2 years later after peaks of phytoplankton development and, hence, peaks of food resources for benthic macroinvertebrates. However, there is no significant correlation between chlorophyll and biomass of macroinvertebrates (including *M*. *affinis*) in the sublittoral zone (Maximov et al., 2021). The results presented here suggest that pelagic production does have effect, but operates primarily through changes in recruitment. This together with density dependence may obscure linear correlation between pelagic and benthic productivity at the studied sublittoral site.

Basically, both limitation by primary production and density‐dependent regulation are interrelated, because the food availability depends naturally not only on sedimentation of pelagic organic matter but also on a number of individuals consuming this food source. Intraspecific competition for food is recognized as most common reason of density‐dependent effects in amphipod populations (Elmgren et al., 2001; Wenngren & Ólafsson, 2002). The idea that density‐dependent factors regulate *M*. *affinis* populations evolved from field studies from the Baltic Sea (Lehtonen & Andersin, 1998; Maximov, 1996; Sarvala, 1986) and then was supported by laboratory experiments (Goedkoop & Johnson, 1994; Hill, 1992; Leonardsson, 1994; Wenngren & Ólafsson, 2002). In inland waters, density‐dependent interactions were found early in Swedish lake Mälaren only, where amphipod growth was negatively correlated with conspecific density and biomass (Johnson & Wiederholm, 1989).

There are reasons to believe that the role of density dependence in regulating the abundance of amphipods is a function of trophic status, being of greater importance in relatively nutrient‐rich water areas, as a rule, with high population density. In the eutrophic Baltic Sea year‐to‐year changes in *Monoporeia* populations seem to be independent from the primary production and can be explained by intrinsic density‐dependent interactions (Lehtonen & Andersin, 1998; Sarvala, 1986); although, primary production, as well as other extrinsic factors (such as temperature, oxygen, structure of fish community) are important explanatory variables for more long‐term changes (Eriksson Wiklund & Andersson, 2014; Olsson et al., 2013; Rousi et al., 2013). In Sweden's large lakes, density‐dependent factors play a marked role in regulating *M*. *affinis* population in the most productive Lake Mälaren, but they are not important in oligotrophic lakes Vänern and Vättern, where variations in amphipod abundance correlate with phytoplankton development (Goedkoop & Johnson, 2001; Johnson & Wiederholm, 1989, 1990). The existence of density‐dependent relationships in oligotrophic Lake Krivoe is likely due to the lesser depth of the studied site (8 m) compared with sites in Sweden's lakes (50 m and more), where powerful hypolimnion isolates the profundal benthic communities from the productive euphotic layer. Also, in the small Lake Krivoe, allochthonous detritus that is transported from the littoral zone and catchment area would be a noticeable source of food for amphipods that is practically absent at offshore sites in Vänern, Vättern, and Mälaren.

Thus, food limitation is a plausible hypothesis for explaining both density dependence and correlation of population recruitment with environmental factors such as concentration of chlorophyll and (at least partly) temperature. The most probable underlying mechanism is intraspecific food competition. The importance of other supposed density‐dependent (predation and other interspecific interactions) factors as a mechanism regulating *M*. *affinis* population in the lake is equivocal. Benthic amphipods are the most important prey items of perch and vendace in Lake Krivoe (Berezina et al., 2018). There are marked seasonal differences in the foraging strategies with distinct prey switching caused by the availability of prey (Berezina et al., 2018, 2021). As noted above, this dietary shift can partly explain the negative correlation between water temperature and recruitment of *M*. *affinis*. Similarly, it seems possible that during the periods of high abundance of *M*. *affinis* fish predators may be feeding selectively on amphipods resulting in density‐dependent mortality. Unfortunately, we have no long‐term data on fish diet as well as on fish stocks in the lake to evaluate how much predation has affected the population of amphipods. From invertebrate predators, *M*. *affinis* can be only consumed by large nectobenthic amphipod *Gammaracanthus loricatus* (Berezina, 2007). However, this species inhabits the profundal zone (depth >20 m) and is absent at the study sublittoral site.

Johnson and Goedkoop (1992) showed that the abundance of *M*. *affinis* and chironomids was negatively correlated in Lake Mälaren, suggesting exploitative competition and/or interference as plausible mechanisms. It was also suggested that amphipods either may passively or actively prey on juvenile chironomids. However, in Lake Krivoe such negative interactions are unlikely to be important, because the population dynamics of *M*. *affinis* was found to be positively correlated with chironomids abundance. The role of chironomids as potential food sources is not substantial enough to explain changes in *M*. *affinis* abundance. Indeed, *M*. *affinis* is a nonselective deposit feeder, which can consume small benthic organisms such as juvenile chironomids, but preferably feeds on newly settled phytoplankton (Aljetlawi et al., 2000; Goedkoop & Johnson, 2001). The role of other taxa in benthic community was negligible (a small percent in total abundance of macrozoobenthos) (Figure 3); therefore, it seems unlikely that interactions with any other benthic species would be anything important.

In conclusion, the study results demonstrate that amphipod recruitment was negatively influenced by both high population density and high water temperature, but positively affected by food availability, defined as chlorophyll *a*. Previous studies showed that the interannual dynamics of primary production in the Lake Krivoe is appreciably controlled by winter meteorological conditions. Consequently, the dynamics of the *M*. *affinis* population to a large extent was determined by climate variations related to year‐to‐year changes in summer temperature and pelagic production combined with density dependence. My findings suggest that density‐dependent factors play a significant role in regulating the *M*. *affinis* populations, and should be taken into account in the environmental assessments. This is due to the fact that this amphipod is unanimously recognized as being sensitive to pollution and other anthropogenic disturbances, and is often used as a bioindicator or test organism in monitoring programs (Berezina et al., 2013; Löf et al., 2016; Sundelin & Eriksson Wiklund, 1998).

## CONFLICT OF INTEREST

None declared.

## AUTHOR CONTRIBUTION

**Alexey A. Maximov:** Conceptualization (lead); Formal analysis (lead); Funding acquisition (lead); Investigation (lead); Methodology (lead); Writing‐original draft (lead); Writing‐review & editing (lead).

## Data Availability

Data are available from the Dryad Digital Repository: https://doi.org/10.5061/dryad.0zpc866z6.
